# Exploratory studies of oral and fecal microbiome in healthy human aging

**DOI:** 10.3389/fragi.2022.1002405

**Published:** 2022-10-20

**Authors:** Xin Zhou, Baohong Wang, Patrick C. Demkowicz, Jethro S. Johnson, Yanfei Chen, Daniel J. Spakowicz, Yanjiao Zhou, Yair Dorsett, Lei Chen, Erica Sodergren, George A. Kuchel, George M. Weinstock

**Affiliations:** ^1^ The Jackson Laboratory for Genomic Medicine, Farmington, CT, United States; ^2^ Department of Genetics and Genome Sciences, University of Connecticut Health Center, Farmington, CT, United States; ^3^ Department of Genetics, Stanford University School of Medicine, Stanford, CA, United States; ^4^ State Key Laboratory for Diagnosis and Treatment of Infectious Diseases, The First Affiliated Hospital, College of Medicine, Zhejiang University School of Medicine, Hangzhou City, China; ^5^ Yale University School of Medicine, New Haven, CT, United States; ^6^ Oxford Centre for Microbiome Studies, Kennedy Institute of Rheumatology, University of Oxford, Oxford, United Kingdom; ^7^ The Ohio State University Comprehensive Cancer Center, Columbus, OH, United States; ^8^ Department of Medicine, University of Connecticut Health Center, Farmington, CT, United States; ^9^ Shanghai Institute of Immunology, Shanghai Jiao Tong University School of Medicine, Shanghai, China; ^10^ UConn Center on Aging, University of Connecticut Health Center, Farmington, CT, United States

**Keywords:** aging, microbiome, saliva, stool, 16S

## Abstract

Growing evidence has linked an altered host fecal microbiome composition with health status, common chronic diseases, and institutionalization in vulnerable older adults. However, fewer studies have described microbiome changes in healthy older adults without major confounding diseases or conditions, and the impact of aging on the microbiome across different body sites remains unknown. Using 16S ribosomal RNA gene sequencing, we reconstructed the composition of oral and fecal microbiomes in young (23–32; mean = 25 years old) and older (69–94; mean = 77 years old) healthy community-dwelling research subjects. In both body sites, we identified changes in minor bacterial operational taxonomic units (OTUs) between young and older subjects. However, the composition of the predominant bacterial species of the healthy older group in both microbiomes was not significantly different from that of the young cohort, which suggests that dominant bacterial species are relatively stable with healthy aging. In addition, the relative abundance of potentially pathogenic genera, such as *Rothia* and *Mycoplasma*, was enriched in the oral microbiome of the healthy older group relative to the young cohort. We also identified several OTUs with a prevalence above 40% and some were more common in young and others in healthy older adults. Differences with aging varied for oral and fecal samples, which suggests that members of the microbiome may be differentially affected by aging in a tissue-specific fashion. This is the first study to investigate both oral and fecal microbiomes in the context of human aging, and provides new insights into interactions between aging and the microbiome within two different clinically relevant sites.

## Introduction

Advanced age is accompanied by inevitable, yet highly variable, declines in physiological function involving different tissues and organs (Lopez-Otin et al., 2013). For most common chronic diseases in adults, such as dementia, cancer and cardiovascular disease, aging represents the single most important risk factor (Percival, 2009). Older adults are also often impacted by factors ranging from social and behavioral to physiologic and biological including altered eating habits, decreased physical exercise (Woodmansey, 2007), diminished gut motility (O'Mahony et al., 2002), declined immune function (Gomez et al., 2005; Shaw et al., 2010), and decreased gut stem cell regenerative capacity (Oh et al., 2014). Collectively, these changes may alter basic biological processes across many different tissues, while influencing and also being influenced by the host microbiome (Tiihonen et al., 2010), which is the community of microorganisms that live on the human body.

A large number of microorganisms colonize the human body and can have profound effects on human health (Group et al., 2009; Human Microbiome Project, 2012; Gao et al., 2022). Human saliva contains 10^8^ to 10^9^ microorganisms per milliliter (Percival, 2009) and the gut harbors at least 3.9 × 10^13^ microorganisms per adult human (Sender et al., 2016). Both salivary bacterial flora (He et al., 2015) and fecal bacterial flora (Henao-Mejia et al., 2012) are closely related to the onset and progression of various diseases (Zhou et al., 2019; Zhou et al., 2020). The disruption of microbiome composition associated with poor health is referred to as dysbiosis (Tamboli et al., 2004). For example, salivary microbiome dysbiosis has been associated with periodontitis (Faveri et al., 2008), respiratory system infections (de Steenhuijsen Piters et al., 2016), and Alzheimer’s disease (Shoemark and Allen, 2015). In contrast, fecal microbiome dysbiosis has been linked to inflammatory bowel disease (Manichanh et al., 2012), frailty (van Tongeren et al., 2005; Claesson et al., 2012) and a higher risk of opportunistic infection (Sekirov et al., 2008).

From a different perspective, bacteria can be used as an intervention for diseases such as opportunistic infection of the gut. Fecal microbiota transplantation represents a novel technique for reconstructing a healthy bacterial community in patients with detrimental gut flora (Aroniadis and Brandt, 2013; Young, 2016). For example, oral administration of a mixed bacterial formula is clinically used in the management of recurrent *Clostridium difficile* infection (Khanna et al., 2016). A more recent study reported certain rejuvenating effects of young microbiota on aged killifish (Smith et al., 2017) and mice (Thevaranjan et al., 2017) following fecal microbiota transplantation. Therefore, understanding the composition of the microbiome in young and older individuals may help explain the elevated frequency of various diseases in old age and provide mechanistic insights that could lead to treatments.

The notion of a healthy gut bacterial community has been largely based on studies in humans and most reports have focused on young adults (Group et al., 2009). Few studies have systematically evaluated the microbiome in the context of disease-free aging; one recent study on the Chinese population (Bian et al., 2017) showed that aging did not affect stool microbiome composition. However, other studies suggest that the microbiome in older adults is less stable than in younger individuals (Claesson et al., 2011; Jeffery et al., 2016). Moreover, several studies have reported that the relative abundance of *Clostridiales* (Biagi et al., 2010; Claesson et al., 2011; Saraswati and Sitaraman, 2014) and *Alistipes* (Claesson et al., 2012; Langille et al., 2014) groups in the microbiome community is greater in aged hosts. Furthermore, since factors other than age, such as being institutionalized, place of residence, and diet, can affect the microbiome (Human Microbiome Project, 2012), it is often difficult to distinguish changes associated with aging *versus* those resulting from varied confounding diseases and factors.

These confounding factors may contribute to the reported inconsistencies in the literature. For example, *Bacteroides*, which is a prominent member of the human gut microbiome, was shown to be elevated in older adults in some studies (Hopkins and Macfarlane, 2002; Claesson et al., 2011) and decreased in others (Woodmansey et al., 2004). It was also recently observed that OTUs in the genus *Bacteroides* change differently with aging in the older microbiome-focused ELDERMET (http://eldermet.ucc.ie) cohorts (Cusack et al., 2013; Jeffery et al., 2016). This indicates that studying the bacterial microbiota composition at the OTU level may be necessary to adequately understand differences between age groups.

We compared the oral and fecal microbiomes of healthy young and older adults. In this manner, we reduced the possible confounding effects of illnesses and drugs on the microbiome. All subjects were community-dwelling and fully independent and frailty was excluded to minimize the confounding effects of frailty, disability, or life in an institutionalized setting. We collected both saliva and stool samples from each subject to determine whether the bacterial microbiota from these tissues follows the same pattern of change during aging.

## Methods

### Subject recruitment

All research was conducted following approval by the University of Connecticut Health Center Institutional Review Board (Number: 14-194J-3). Following informed consent, the oral and fecal microbiome samples were obtained from 10 healthy young (HY, 23–32; mean = 25 years old) and 13 healthy old (HO, 69–94; mean = 77 years old) volunteers residing in the Greater Hartford, CT, United States of America region using services of the UConn Center on Aging Recruitment and Community Outreach Research Core (http://health.uconn.edu/aging/research/research-cores/). Recruitment criteria were established to select healthy adults experiencing “normal aging” who are reflective of the typical health conditions of the population within the corresponding age groups (Stephanie Studenski and Resnick, 2009). Selecting this type of cohort increases the generalizability of our studies and the likelihood that these findings can be translated to the general population (Stephanie Studenski and Resnick, 2009). Subjects were carefully screened to exclude potentially confounding diseases and medications. Individuals who reported chronic or recent (i.e., within 2 weeks) infections were also excluded. Subjects could have chronic diseases but were excluded if the following were present: congestive heart failure, kidney disease (serum creatinine >1.2 mg/dl in men and >1.1 mg/dl in women), diabetes mellitus requiring medications, use of antibiotics, immunosuppressive disorders or the use of immunosuppressive agents including oral prednisone in doses >10 mg day. Since declines in self-reported physical performance are highly predictive of frailty and subsequent disability and mortality (Hardy et al., 2011), all subjects were questioned as to their ability to walk. For those who self-reported an inability to walk (Hardy et al., 2011), the “Timed Up and Go” (TUG) test was performed and measured as the time taken to stand up from the sitting position, walk 10 feet and return to sitting in a chair (Podsiadlo and Richardson, 1991). A TUG >10s score was considered an indication of increased risk of frailty and resulted in exclusion from the study (Rockwood et al., 2000).

### Sample collection

Fecal samples were collected using the Fisherbrand™ Commode Specimen Collection System (Thermo Fisher Scientific, Waltham, MA, United States). Saliva samples were collected by asking each subject to let saliva collect in their mouth for at least 1 min. The subject was then asked to drool into a labeled 50 ml collection tube (Falcon, sterile conical polypropylene tube with flat-top screw cap). This process was repeated multiple times to collect larger volumes of saliva (2–5 ml). Oral cavity microbiome samples were from the dorsum of the tongue using Catch-All™ Sample Collection Swabs and swabbing 1 cm^2^ of the center of the tongue for 5 s. Immediately after swabbing, each swab was swirled in MO BIO’s PowerSoil DNA Isolation Kit (Mo Bio Laboratories, Carlsbad, CA, United States) collection tube with 750 *ul* prefilled collection buffer (Tube C1). The swab sponge was pressed against the tube wall multiple times for 20 s to ensure the transfer of bacteria from the swab to the solution. The specimen in the collection tube was kept cold until ready for processing.

### DNA sequencing

Fresh samples were stored at -80 °C immediately after collection for the microbiome analysis. Total DNA was extracted from fecal samples using the Power Soil DNA Extraction kit (Mo Bio Laboratories, Carlsbad, CA, United States) according to manufacturer’s protocol. Bacterial 16S rRNA gene DNA was amplified using the 27F/534R primer set (27F 5′-AGA​GTT​TGA​TCC​TGG​CTC​AG-3′, 534R 5′-ATT​ACC​GCG​GCT​GCT​GG-3′). PCR reactions were performed using Phusion High-fidelity PCR Master mix (Invitrogen, Carlsbad, CA, United States) at the following conditions: 95 °C for 2 min (1 cycle), 95 °C for 20 s/56 °C for 30s/72 °C for 1 min (30 cycles). PCR products were barcoded and purified using Agencourt AMPure XP beads (Beckman coulter, Brea, CA, United States) according to manufacturer’s protocol. Libraries were prepared with Illumina’s protocol for the MiSeq platform. DNA sequencing was conducted on an Illumina MiSeq system.

### Sequencing data analysis

Raw reads were filtered according to the sequence length and quality. Filter-pass reads were assembled using Flash assembly software, where the minimum overlap requirement is 30 bp and the maximum mismatch ratio is 10% (Magoc and Salzberg, 2011). After assembly, chimeric sequences were removed using the USEARCH software based on the UCHIME algorithm (Edgar et al., 2011). After the barcode was removed from each sequence, operational taxonomic units (OTUs) were calculated using a *de novo* OTU picking protocol with a 95% similarity threshold (specifically, a cluster at 98% similarity first, and then cluster the output at 95% similarity as suggested by USEARCH http://www.drive5.com/usearch/manual/uparse_otu_radius.html). The taxonomy assignment of OTUs was performed by comparing sequences to the Ribosomal Database Project (RDP) (Wang et al., 2007) at https://rdp.cme.msu.edu/with a cutoff of 0.8 (Claesson et al., 2009). A total of 599,211 assembled reads were generated from 46 samples with a mean read depth of 13,026 and a standard derivation of 6,099. The read depth ranged from 3,700 to 29,332. Multivariate trees were plotted using Tree Of Life v1.0 (Letunic and Bork, 2007; Letunic and Bork, 2011). To normalize the sequence depth of each sample, 3,700 reads were randomly picked from each sample for alpha diversity analysis, and the statistical significance of differences were calculated using the unpaired *t*-test with Welch’s correction. The R package “Phyloseq” was used for alpha diversity and beta dissimilarity analysis (McMurdie and Holmes, 2013). The two-sided Student’s *t*-test was used for significance testing for normally distributed variables, otherwise the Mann-Whitney *U* test was used for significance testing. Linear discriminant analysis based on effect size (LEfSe) (Segata et al., 2011) was performed with the web tool at http://galaxyproject.org/. The statistical tests were completed with R packages “plyr” and “ggplot2” (Wickham, 2009). Power estimation was performed using the R package “pwr.” Plots were created with GraphPad Prism version 6.00 for Mac (GraphPad Software, La Jolla, California, United States of America).

## Results

### Overview of the study dataset

Oral and fecal microbiomes were surveyed by collecting saliva and stool samples, respectively, from each of the 23 subjects recruited for this study, which included 10 young individuals (ages 23–32) and 13 older adults (ages 69–94) (Table 1). Total DNA was extracted from the samples, and the 16S rRNA gene was amplified and subjected to DNA sequencing. A total number of 548 OTUs were generated as described in the methods. OTUs with less than five reads were excluded from the dataset. Saliva samples yielded 381 OTUs, whereas stool samples yielded 405 OTUs. In addition, saliva samples had 120 young-related OTUs and 35 aged-related OTUs, whereas fecal samples contained 39 young-related OTUs and 150 aged-related OTUs (Table 2).

**TABLE 1:** Subjects characteristics.

Groups	Young (n = 10)	Old (n = 13)
Age (year range)^a^	25+-3.20 (23–32)	77 ± 6.16 (69–94)
Male/female	2/8	7/6
Body mass index^a^	24.98+-4.77	27.43 +- 3.16

^a^ Data are expressed as the mean ± SE.

**TABLE 2:** Summary of OTU selected by each method.

	Saliva	Stool	Total number	
Total OTU number	381	405	548	
Total Read count	331,977	267,234	599,211	
	**Young**	**Old**	**Young**	**Old**
Age-associated OTU	120	35	39	150
Age-associated reads	20,038	21,189	60,876	26,232
Binary OTU	103	27	24	131
Binary reads	3,105	219	1,657	11,866
LEfSe	29	2	13	24
LEfSe reads	16,331	16,627	60,262	10,584
Random Forest (RF)	23	7	12	18
RF reads	7,513	5,120	7,926	8,806

Operational taxonomic unit (OTU) was calculated using a *de novo* OTU picking protocol with a 95% similarity threshold.

### Bacteria flora richness in the young and aged cohort

The diversity of bacterial flora is a crucial aspect of the microbiome (Human Microbiome Project, 2012). The richness (the total number of distinct bacterial taxonomy present) and diversity (the variation in the number of different bacterial types) were computed for each sample by treating each OTU as a taxonomy.

**FIGURE 1:**
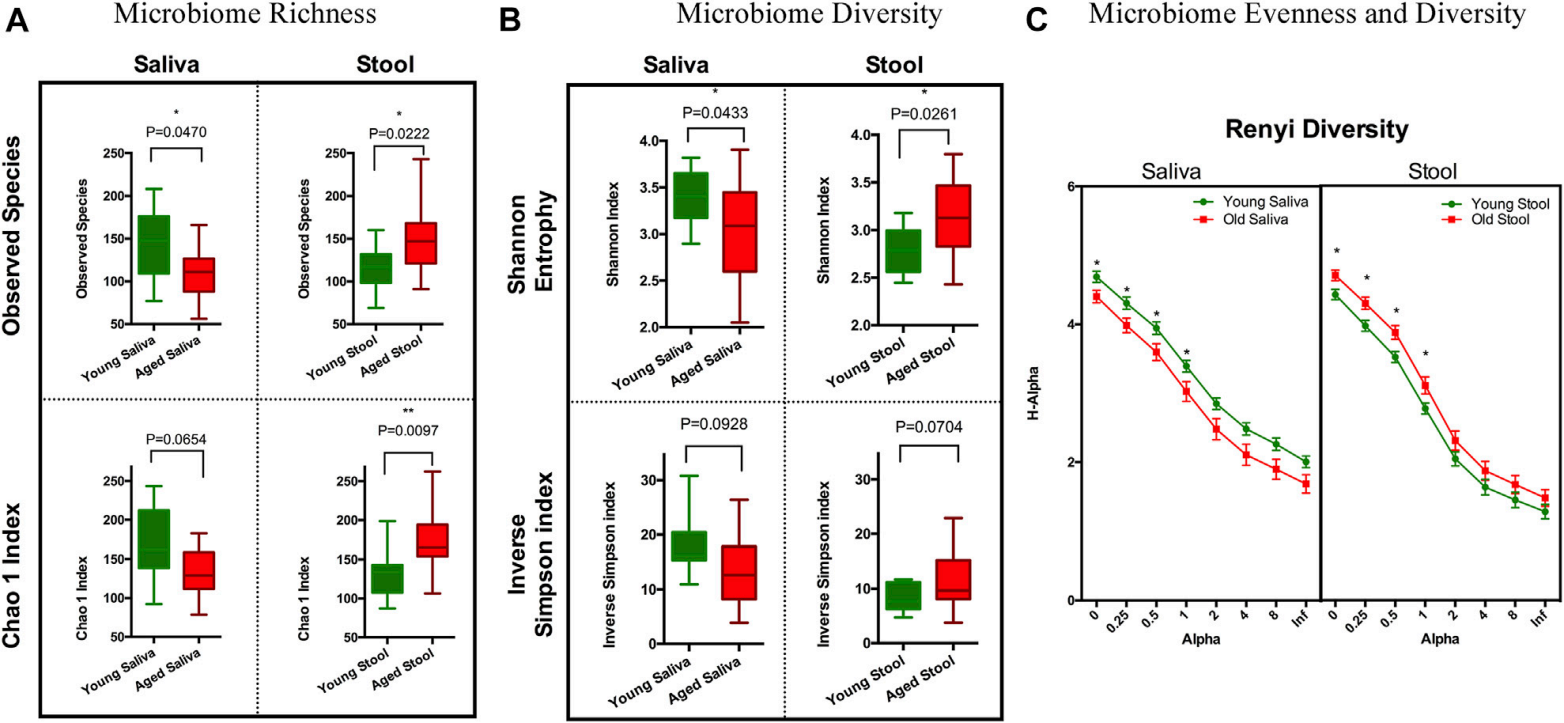
Ecology richness and diversity of the microbiome. **(A)** Microbiome richness plot of a saliva and stool sample (*p < 0.05; **p < 0.01). **(B)** Microbiome Shannon diversity and inverse Simpson index plot of a saliva and stool sample. **(C)** Renyi diversity of the microbiome in different age groups. *X*-axis represents the alpha-value (increased alpha-value indicates a higher weight on dominant organisms when calculating diversity); *Y*-axis is the diversity index calculated based on each alpha-value (*p < 0.05. *t*-test with Holm-Sidak correction).

To estimate species richness, we analyzed our data using the Observed Species and Chao1 index (Hughes et al., 2001). The number of species observed in saliva samples from the old cohort were significantly lower than that from the young cohort. Although less pronounced, a similar trend was observed when calculating the Chao1 index. Stool from the old cohort showed a higher mean value on Observed Species compared to the young cohort, and the Chao1 index showed a similar trend. We think this is because singletons and doubletons contribute more to the total variance in stool than saliva (Figure 1A). Next, we applied Shannon entropy and the inversed Simpson index to measure the ecological diversity of the bacterial community. Like the observations we made for species richness, the Shannon entropy of saliva from the old cohort exhibited a lower value compared to the young cohort, and there was a reversed pattern for the stool sample (Figure 1B). The inversed Simpson index provides a higher weight on more abundant taxonomies than the Shannon entropy. This is another piece of evidence showing that the bacterial population difference between young and aged cohort resides in less abundant organisms within the bacterial microbiota.

To further understand these differences in diversity and perform a comprehensive comparison regarding the nature of microbial ecology in these subjects, we plotted the Renyi diversity (Li et al., 2012) of microbial populations for young and aged cohorts per body sites. When the alpha-value of the Renyi diversity grows, a higher mathematical weight was assigned to taxa with higher relative abundance. The slope of the curve shows the general evenness of the microbial community, i.e., a small curve slope indicates high evenness of the community (Figure 1C). Comparable to the aforementioned tendencies, the importance of differences in diversity is less when the alpha-value is increased, which indicates that the bacterial community has more similarity when just the most numerous species are evaluated. This again proves that the difference between young and old cohorts is caused by non-dominate organisms.

### Characterization of the taxonomic properties within the microbial community

To comprehend the organization of bacterial communities in terms of the main taxonomies, we constructed a hierarchical clustering tree based on the 20 most abundant OTUs from all 46 samples (Supplementary Figure S1A). As expected, the saliva and stool samples clearly formed two distinct main branches. However, the saliva sample from subject #107 also contained representative OTUs found in stool samples, whether this is the true microbiome composition of this subject or reflects severe reflux or sample contamination occurring during or following sample collection is unknown. We did not notice an extra branch for age factors among the top 20 OTUs. This suggests that the dominant bacterial microbiome is largely stable throughout the aging process in healthy humans.

Next, we examined the beta dissimilarity based on all available OTUs under the unweighted UniFrac Distance (Lozupone et al., 2007). This method employs a beta dissimilarity calculation based on phylogenetic trees. According to the Principal Coordinate Analysis (PCoA) plot, the stool bacterial microbiome community structure from older adults seemed different from younger individuals (PERMANOVA: Pr>(F) value = 0.047). The salivary bacterial microbiome community structure from the aged cohort is fairly similar to that in the young cohort (PERMANOVA: Pr>(F) value = 0.519) (Supplementary Figure S1B). In conjunction with the results of the cluster-based analysis, we infer that the bacterial type and abundance of the microbiome population as a whole may not vary much with age. As a benefit of our unique design, we can also compare the pairwise dissimilarity of the saliva and stool microbiome between age groups. Consistent with the PREMANOVA result, the overall dissimilarity of the stool microbiome between groups is significantly higher than that of the saliva microbiome (Supplementary Figure S1C). However, the saliva sample shows a higher populational level dissimilarity in the older population compared with the younger population, and this trend is not obvious among stool samples (Supplementary Figure S1D).

### The abundance of bacteria taxonomy changes with age

**FIGURE 2:**
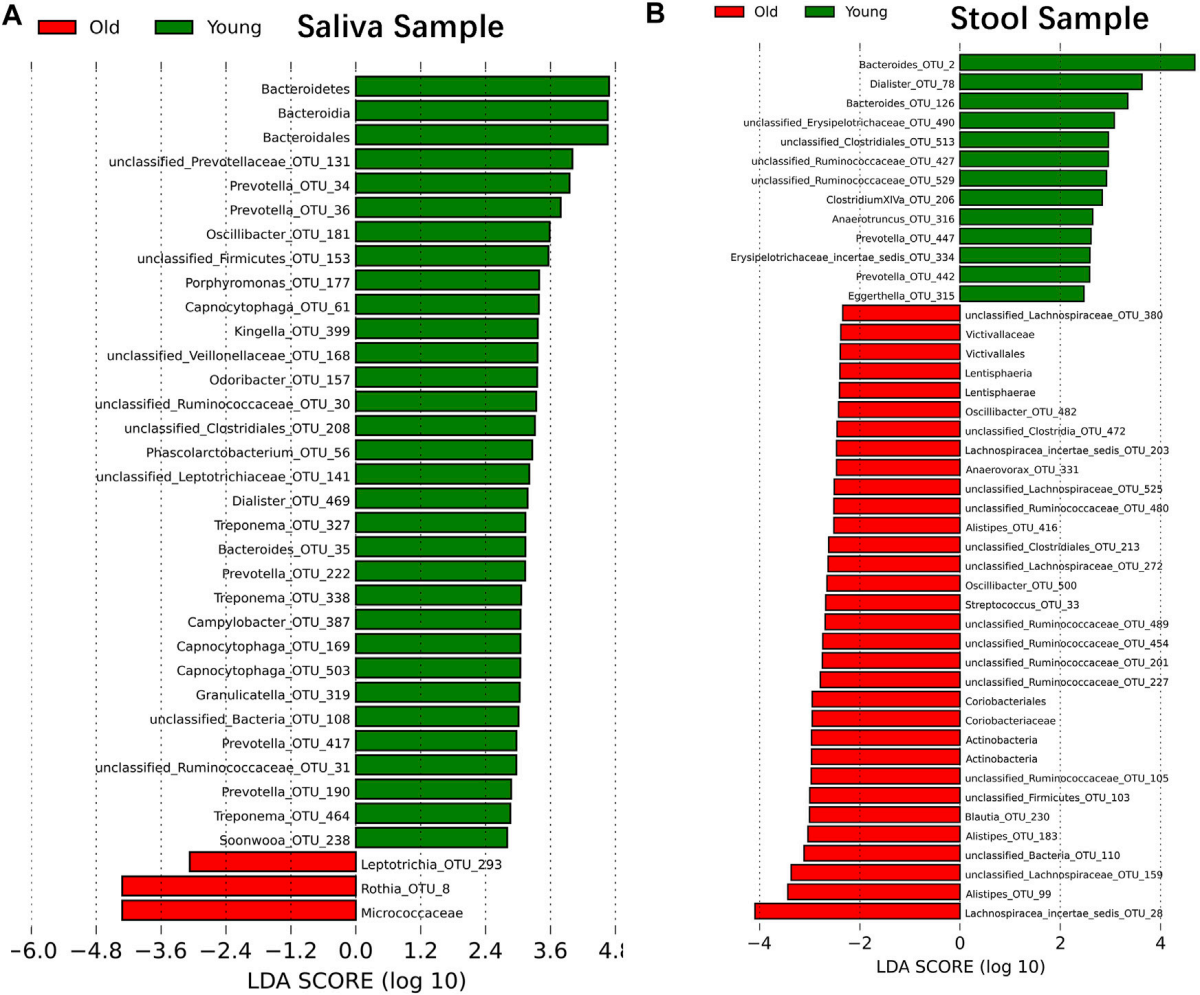
Microbiome modulated by age. The bacteria taxonomies changed with age. Data are plotted with a linear discriminant analysis score with log10 transformation. Green bars indicate this taxonomy is more abundant in young cohorts and red bars indicate this taxonomy is more abundant in aged cohorts. **(A)** Saliva sample. **(B)** Stool sample.

Although samples from the two age cohorts do not form distinct branches, there is evidence from ecological diversity estimates that some taxa may have a varied distribution of abundance between cohorts. To calculate the taxa that vary across the two cohorts, we performed a linear discriminant analysis based on effect size (LEfSe) (Segata et al., 2011). We identified 29 OTUs that are statistically more abundant in the young cohort and two OTUs in the older group within saliva samples at a significance level of 0.05 for the factorial Kruskal–Wallis test. Also, the order of *Bacteroidales*, the class of *Bacteroidia* and the phylum of *Bacteroidetes* is more abundant in the young cohort, and the family of Micrococcaceae is more abundant in the older group (Figure 2A). For stool samples, we were able to identify 13 OTUs that are more abundant in the young cohort and 24 OTUs that are more abundant in the older group. Besides single OTUs, there are two clusters that are higher in aged populations. One cluster is the order *Coriobacteriales*, and consequently, the family Coriobacteriaceae, the phylum *Actinobacteria* and the class *Actinobacteria*; another cluster is the Victivallaceae family, order *Victivallales*, class *Lentisphaeria* and phylum *Lentisphaerae* (Figure 2B).

Since LEfSe has the potential to miss OTUs with a lower frequency, we applied two additional approaches to identify taxonomies with a distinct distribution across groups. Random Forest (Breiman, 2001) is one of the most accurate machine learning methods for categorization of factor variables and is one of the most popular classification techniques (Knights et al., 2011; Statnikov et al., 2013). It generates an importance score for each variable that represents how accurate this variable is to distinguish groups. We trimmed OTUs that have global variables less than 0.005 in each site to remove the background, and consequently, 258 OTUs are left in the stool samples and 155 OTUs are left in the saliva sample. Then, we generated a list of 30 OTUs with the highest importance value (Supplementary Figure S2).

For samples with a low abundance that nonetheless show a distinct distribution between groups, Random Forest may give a low importance value since its controlled classification calculation is based on the overall sample. To compensate for this, we additionally identified the OTUs that were present in one age cohort but lacking in another (independent of their abundance) and we named them “Binary” OTUs. Among all saliva samples, the young population has 103 binary OTUs and the older population has 27. Among stool samples, the young population has 24 binary OTUs and the older population has 131 (Table 2). A Venn Diagram shows the relationship of three methods and is provided in Supplementary Figure S4.

By combining the three procedures described above, we were able to identify 155 OTUs from 381 OTUs in saliva samples and 189 OTUs from 405 OTUs in stool samples (Table 2). We consider these OTUs bacteria taxonomies altered with age. These readings correspond to OTUs that occupy roughly 10% of saliva samples and 30% of stool samples in terms of the relative abundance and despite comprising around 50% of OTUs. Also, age-related OTUs show an age-associated distribution (Supplementary Figure S3) as the young cohort seems to have a higher percentage of young cohort-related OTUs as does the aged cohorts. We also investigated the possibility of microbiome translocation as implicated in several previous reports. We found that the six most abundant saliva taxa are relatively higher (W = 38, *p* = 0.10) in the stool microbiome of old individuals (median relative abundance = 0.0832%) compared to young individuals (median relative abundance = 0.0357%), which indicates a trend of the saliva microbiome migrating to the gut (Supplementary Figure S7). This translocation effect was not obvious in the saliva microbiome, and the young and old cohort have a similar median relative abundance (young median relative abundance: 0.0499%, old median relative abundance: 0.145%, W = 61, *p* = 0.83) of stool major microbiome in their saliva samples.

**FIGURE 3:**
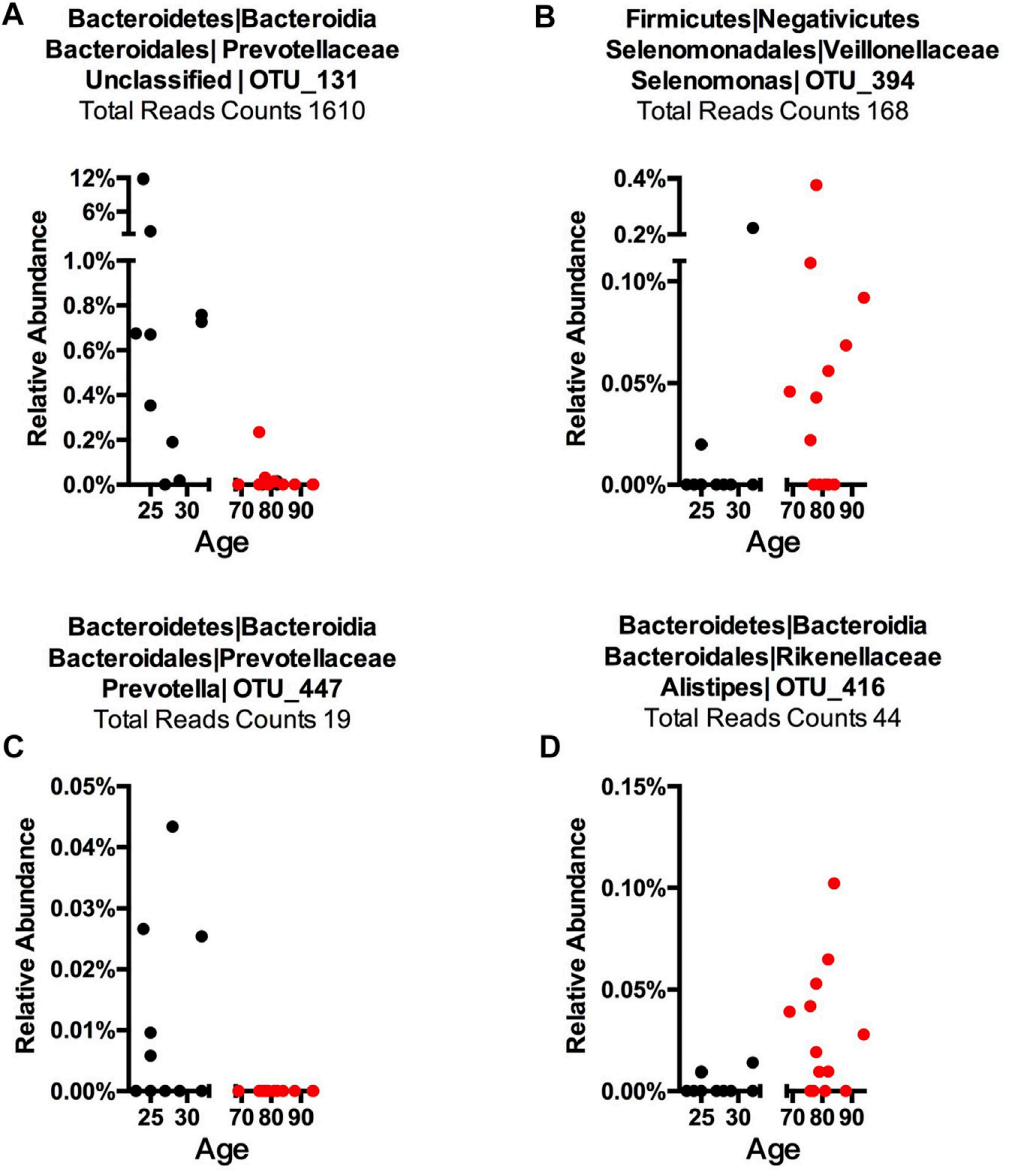
Representative OTUs for each Core Microbiome. The relative abundance of each of the OTUs is plotted. **(A)** Young cohort representative OTU in a saliva sample, **(B)** aged cohort representative OTU in a saliva sample, **(C)** young cohort representative OTU in a stool sample, and **(D)** aged cohort representative in a stool sample. Each dot represents one subject with the *X*-axis showing this subject’s age and the *Y*-axis showing the percentage of reads of the OTU that belongs to each sample. The total reads across sample and subject are listed. The title shows phylum, class, order, family, genus, and OTU number, while “Unclassified” indicates this OTU could only be classified at a higher taxonomy rank.

To detect age-related OTUs with increased statistical certainty and provide a reference to similar studies in the future, we performed a power analysis of the presence/absence study. We found that OTUs that were present in at least 40% of each age-group were statistically more reliable (Supplementary Figure S8). Thus, we applied this criterion to our “Binary” OTUs when combining the three methods. Among the 155 age-related OTUs in saliva, 120 were more abundant or only present in young cohorts while 35 OTUs occurred in the older group (Table 2). For young-related OTUs, 10 were picked up by all three methods, which belong to the genus *Prevotella*, *Soonwooa*, *Treponema*, *Campylobacter*, *Dialister* and *Capnocytophaga* (Supplementary Figure S4). A total of 43.06% of the reads in 23 saliva samples from a young cohort belong to the genus *Prevotella*, followed by *Unclassified* Prevotellaceae (12.89%) and *Capnocytophaga* (7.53%) (Supplementary Figure S5A). For advanced age-related OTUs in saliva samples (Supplementary Figure S5B), *Rothia* occupies 81.15% of entire advanced age-related population, followed by *Capnocytophaga* (10.21%) and *Prevotella* (3.37%). We also noticed a group of bacteria that were prevalent (at least 40%) in one cohort and showed an obvious distribution associated with aging (Figure 3 and Supplementary Figure S6). For example, OTU_197 (Supplementary Figure S6B) *Tannerella* was more prevalent and abundant in aged saliva samples than in young saliva samples. Given the fact that *Tannerella forsythia* and *Treponema denticola* are among three major pathogens involved in periodontitis (Scapoli et al., 2012), this could be evidence that the oral cavity environment is more prone to *Tannerella* growth when aging. Moreover, OTU_190 (Supplementary Figure S6A) belongs to a species of *Prevotella*. This OTU could only be found in young saliva cohorts and was only present in 164 reads in the entire sequence. The same pattern was found in stool samples for OTU_447 (Supplementary Figure S6C). The loss of these *Prevotella* species may not affect the whole *Prevotella* population in terms of overall *Prevotella* abundance, but it is very interesting as to why a specific species could appear or disappear during the aging process.

**FIGURE 4:**
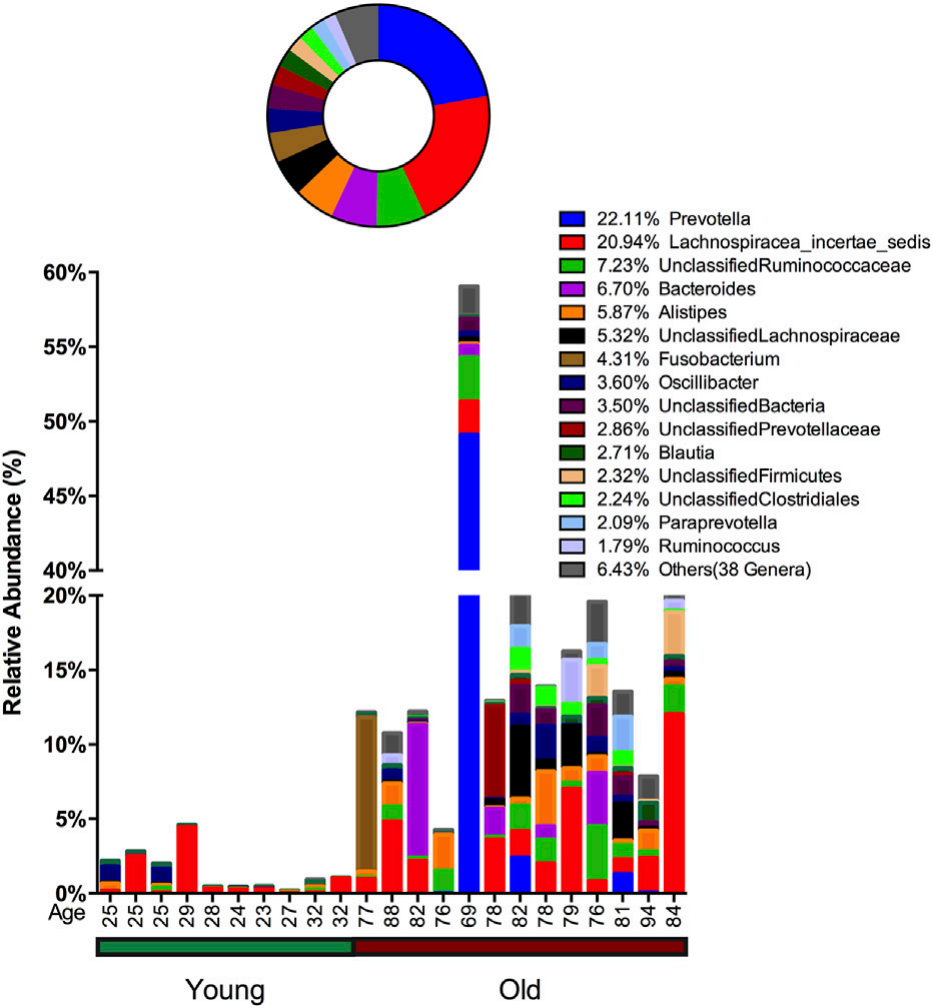
Advanced age-related microbiome in the stool sample. The advanced age-related stool microbiome population consists of 150 OTUs belonging to 53 genera with the 15 most prevalent taxa given below and the remaining 38 genera merged and shown as “Others.” The abundance percentage is determined by dividing the number of reads for each OTU by the total number of reads for this sample. The circle plot shows a combined percentage plot, which is the total of the abundance percentage of each species across all 23 individuals.

In stool samples, 39 OTUs are more abundant or only present in young cohorts and 150 OTUs occur in old cohorts (Figure 4 and Supplementary Figure S5). Among the young cohort associated microbiome (Supplementary Figure S5C), 85.12% belong to *Bacteroides*. This is consistent with several previous reports (Bartosch et al., 2004; Woodmansey et al., 2004; van Tongeren et al., 2005). For the 10 most dominant genera of the young cohort-related stool microbiome, half belong to the order *Clostridiales*, which indicates that the order *Clostridiales* may be sharply affected by age. *Dialister* has been previously reported as less abundant in the over 70-year-old population in Korea (Park et al., 2015), and the abundance of this genus is also believed to correlate with aging (Biagi et al., 2010). Among the 53 genera that are more abundant in the aged cohort (Figure 3), Lachnospiraceae *Incertae Sedis* and *unclassified* Lachnospiraceae contribute to 26.26% of the entire population. 6.7% belong to *Bacteroides*, 5.87% of this core population is *Alistipes*, and has been reported to be more abundant in the aged mouse gut (Langille et al., 2014) and associated with the more frail aging population in Europe (Claesson et al., 2012). It is worth noting that among 150 OTUs that are more abundant in the aged stool population, 7.23% of them are *unclassified* Ruminococcaceae (0.56% in young cohorts), 2.24% are *unclassified Clostridiales* (0.35% in young cohorts), 3.5% are *unclassified Bacteria* and 2.86% are *unclassified* Prevotellaceae.

## Discussion

Bacterial microbiota changes associated with aging were described decades ago (Knights et al., 2011) but given the presence of potentially confounding factors, such as varied chronic diseases, frailty, medications, or institutionalization, it has not been possible to discern microbiome changes attributable to normal aging processes. Nevertheless, it is proposed that such age-associated microbiome changes could contribute to chronic inflammation, which represents one of the hallmarks of biological aging (Scapoli et al., 2012). Our study is, to the best of our knowledge, the first report of combined stool and saliva microbiome analysis conducted in the context of healthy aging. Despite a small sample size and low statistical power (Supplementary Figure S9), we nonetheless observed that several characteristics of saliva and the stool microbiome were different between aged healthy individuals and young controls. Moreover, the opportunistic pathogenic genus was found to be enriched in both aged saliva and fecal samples, such as members from the genera *Rothia*, *Mycoplasma*, *Selenomonas, Tannerella*, and *Alistipes.* More importantly, by combining three comparison methods, we found that the composition of predominant bacteria in saliva were relatively similar, but slightly different in stool between healthy aged and young people, which indicated that the dominant bacteria communities were not significantly affected by age. This is the first study investigating age-related changes in the saliva and stool microbiomes among the same carefully selected healthy cohorts. Our results indicate that the dominant bacteria might play an important role in maintaining human health status in the aging process.

Before the age of next generation sequencing, several changes in aged people were revealed using culture methods (Hopkins et al., 2001) and q-PCR based 16S rRNA gene screening (He et al., 2003). Recently, the application of next generation sequencing of 16S rRNA genes has allowed the study of the bacterial microbiome in a more comprehensive manner. Employing this technique, several reports focusing on the European populations have shown that the microbiome compositional difference is affected by age, lifestyle, and geographical location (Claesson et al., 2011; Claesson et al., 2012; Yatsunenko et al., 2012). Some reports indicate that decreased gut flora richness and diversity are associated with hospitalization and antibiotic treatment (Bartosch et al., 2004), which could be a signature of dysbiosis (Langille et al., 2014; O'Toole and Jeffery, 2015). We present this study as a novel investigation focusing on understanding the community of living individuals who were all in relatively good health. In our study, the gut microbiome of the aged cohort indicated a trend toward higher ecological richness and diversity. This age-associated bacteria enrichment has been previously reported in *Drosophila melanogaster* (Ren et al., 2007). In antibiotic-free older adults, increased counts of facultative anaerobes have been reported (Woodmansey et al., 2004). We believe that this increased gut microbiome diversity in aged cohorts could be ascribed to certain newly emerged bacteria or from the decline of some inhibiting factors such as age-associated intestinal immune function decline (immunosenescence) (Franceschi et al., 2000; Ostan et al., 2008). Certain bacteria groups, such as the *Clostridiales* subpopulation, are considered detrimental in aging-related bacteria composition change (O'Toole and Jeffery, 2015), and we found nine species belonging to *Clostridiales* that are more abundant in the gut microbiota of aged cohorts with at least a 50% prevalence.

Besides the gut microbiota population, we noticed that the aged oral microbiome shows decreased diversity among the same population. We think that several factors may be contributing to this observation. This could be a result of the different local immune system against bacteria in the gut and oral environment (Back et al., 2007; Percival, 2009) that may shape the microbiota in different ways. Of note, saliva represents an oxygen-rich environment, whereas the gut harbors more anaerobic bacteria. Hence, the opposite trends of the saliva and stool microbiome that we observed in the aged population may result from increased oxidative stress. In saliva samples, we observed a decrease of the anaerobic bacteria *Prevotella* and Unclassified Prevotellaceae and an increase of aerobic bacteria *Rothia*. Also, the oral cavity represents a very divergent environment, and the decreased bacterial population and decreased diversity may be associated with a loss of teeth and Xerostomia, both of which are very common in the aged population (Petersen and Yamamoto, 2005).

There are also other confounding variables, such as diet, tobacco use, and alcohol use, that may contribute to changes in the diversity of the gut and saliva microbiome. It was previously found that smoking can lower gut (Opstelten et al., 2016; Gui et al., 2021) and saliva (Jia et al., 2021) microbiome diversity and contribute to the dysbiosis (Wu et al., 2016; Huang and Shi, 2019) in these two body sites. Alcohol usage has also previously been closely linked to decreased gut microbiome diversity (Bajaj, 2019; Philips et al., 2022) and increased oral (Fan et al., 2018; Liao et al., 2022) microbiome diversity. Additionally, alcohol usage is associated with the growth of pathobionts and they can have a long-term effect on the microbiome (Vetreno et al., 2021; Day and Kumamoto, 2022). These cofounding factors should be covered in follow-up studies to create a more comprehensive understanding of their role in the microbiome and healthy aging.

In this study, we did not see dramatic changes of OTUs in older adults’ saliva microbiome compared to young adults. However, we did notice minor changes for some OTUs. For instance, increasing one *Mycoplasma* OTU (OTU_128) could be an indicator that the unbalanced oral community among older adults may create higher tolerance for pathogenic bacteria species, such as *Mycoplasma pneumoniae*, to establish colonization, which is in line with widely reported *Mycoplasma pneumoniae* infection among elderly adults (Miyashita et al., 2008; Takahashi et al., 2009; Parrott et al., 2016). Also, an increased abundance of the genus *Rothia* in older adults has been positively associated with aging and increased pneumonia recently (de Steenhuijsen Piters et al., 2016). In the oral microbiome, this genus is also associated with periodontal diseases (Kim and Reboli, 2015). Thus, the salivary microbiome community changes when aging may also help explain increased pneumonia and periodontal disease frequency among the aged population (Huttner et al., 2009; Kline and Bowdish, 2016). One study focusing on the oral microbiome indicated that patients with periodontitis (Sender et al., 2016) show low saliva bacteria ecological diversity due to the overgrowth of *Selenomonas* and *Streptococcus*. These imbalances in bacterial ecology may contribute to the inflammatory status of periodontitis. We noticed one species of *Selenomonas* (OTU_394) present in eight of thirteen aged cohorts but only two young cohorts along with the newly emerged *Tannerella* species as mentioned above; these bacteria found in aged cohorts and associated with periodontitis strongly suggest a relationship between the saliva microbiome and the emergence of periodontitis (Faveri et al., 2008; Scapoli et al., 2012).

Additionally, the genus *Bacteroides* is dominant among the young cohort-related to the microbiome of stool samples, which is consistent with several previous reports (Bartosch et al., 2004; Woodmansey et al., 2004; van Tongeren et al., 2005). The proportion of the genus *Bacteroides* in the advanced age-related microbiome is decreased, which is possibly associated with an increase of other unclassified bacteria. Inconsistent with our results, the *Bacteroides-Prevotella* group was also found to be decreased in the aged population as well as hospitalized patients (Saraswati and Sitaraman, 2014). Of note, not all differences associated with age are cross-phylum. In the replacement of *Bacteroides,* three elderly individuals showed an increase of *Prevotella*, which can also be proinflammatory (Scher et al., 2013; Moreno, 2015) under certain conditions. Additionally, the persistence of inflammatory stimuli over time represents the biologic background favoring a susceptibility to age-related diseases/disabilities (Franceschi et al., 2000). Moreover, we identified three species of *Alistipes* showing high prevalence and abundance in the aged population. Interestingly, *Alistipes* has been associated with aging in humans (Claesson et al., 2012) and mice (Langille et al., 2014). In a human study, this *Alistipes* genus has been associated with a long stay in medical facilities and increased frailty (Claesson et al., 2012). The microbiome has been reported as an indicator of health conditions (Yoshizawa et al., 2013). Although the mechanism by which these disease indicators come to exist in saliva or stool has not been fully explained, these findings suggest that the microbiome may represent a significant source of discriminatory biomarkers of age-related diseases.

The majority of the age-related microbiome that we identified as being different in healthy older adults represents a minor group that occupies a small percentage of the whole microbiome community. Several recent reports pointed out that single species of bacteria, rather than genus or higher taxonomies, could have a specific character that regulates the immune system or modulates lifespan (Nakagawa et al., 2016; Rossi et al., 2016). We’d like to re-emphasis that we found predominant bacteria that were relatively stable in saliva and in stool between healthy older and young people. These findings indicate that the dominant bacteria phylogenies are relatively stable across the healthy human aging process. In agreement with our study, the stool microbiota of centenarians is dominated by *Bacteroidetes* and *Firmicutes,* which account for 93% of total bacteria (Biagi et al., 2010). Obvious changes can be found more frequently in the composition of dominant bacteria between healthy controls and people with diseases or pathological status. For example, the proportions of the phylum *Firmicutes* and class *Clostridia* were significantly reduced in the diabetic group (Franceschi et al., 2000). Other human intestinal dysbiosis has been demonstrated in subjects with diseases such as obesity (Turnbaugh et al., 2006; Turnbaugh et al., 2009), metabolic syndrome (Vrieze et al., 2012; Murphy et al., 2013), diabetes (Larsen et al., 2010; Qin et al., 2012), and cardiovascular diseases (Wang et al., 2011). Thus, the relatively stable and dominant microbiota in our aged cohort implies that dominant bacteria might play an important role in maintaining human health status. It is worth considering that the stability of the “dominant bacteria” might be a biomarker to assess the health status of aged cohorts.

## Data Availability

The datasets presented in this study can be found in online repositories. The names of the repository/repositories and accession number(s) can be found below: NCBI BioProject ID: PRJNA865362.
