# Supplementary figures and images for: Exploratory studies of oral and fecal microbiome in healthy human aging
Source: Front Aging. 2022 Oct 20;3:1002405. doi: 10.3389/fragi.2022.1002405 (PMC9631447; doi:10.3389/fragi.2022.1002405)

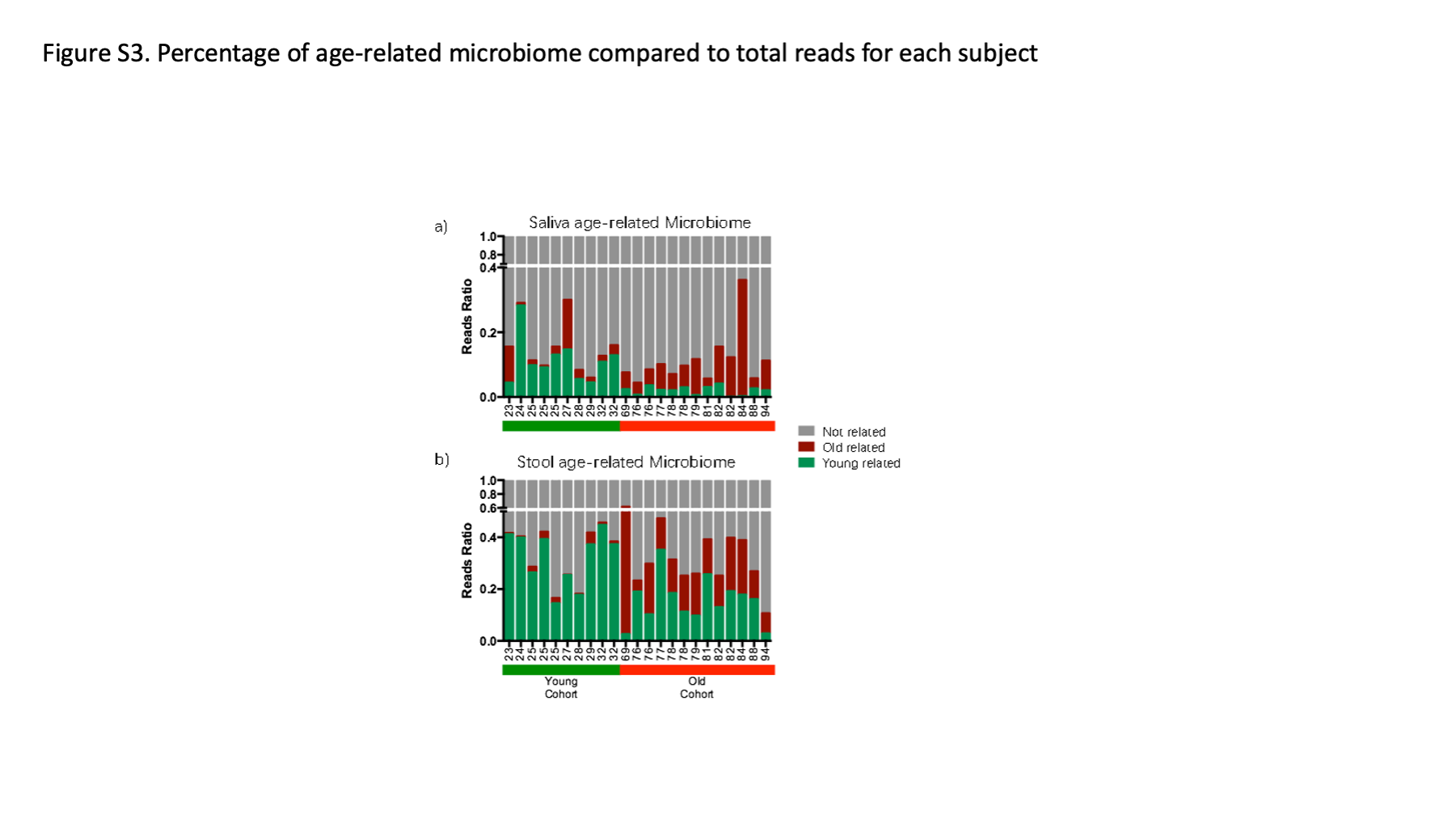

Supplement: Supplementary file 1 [file Image3.tiff]

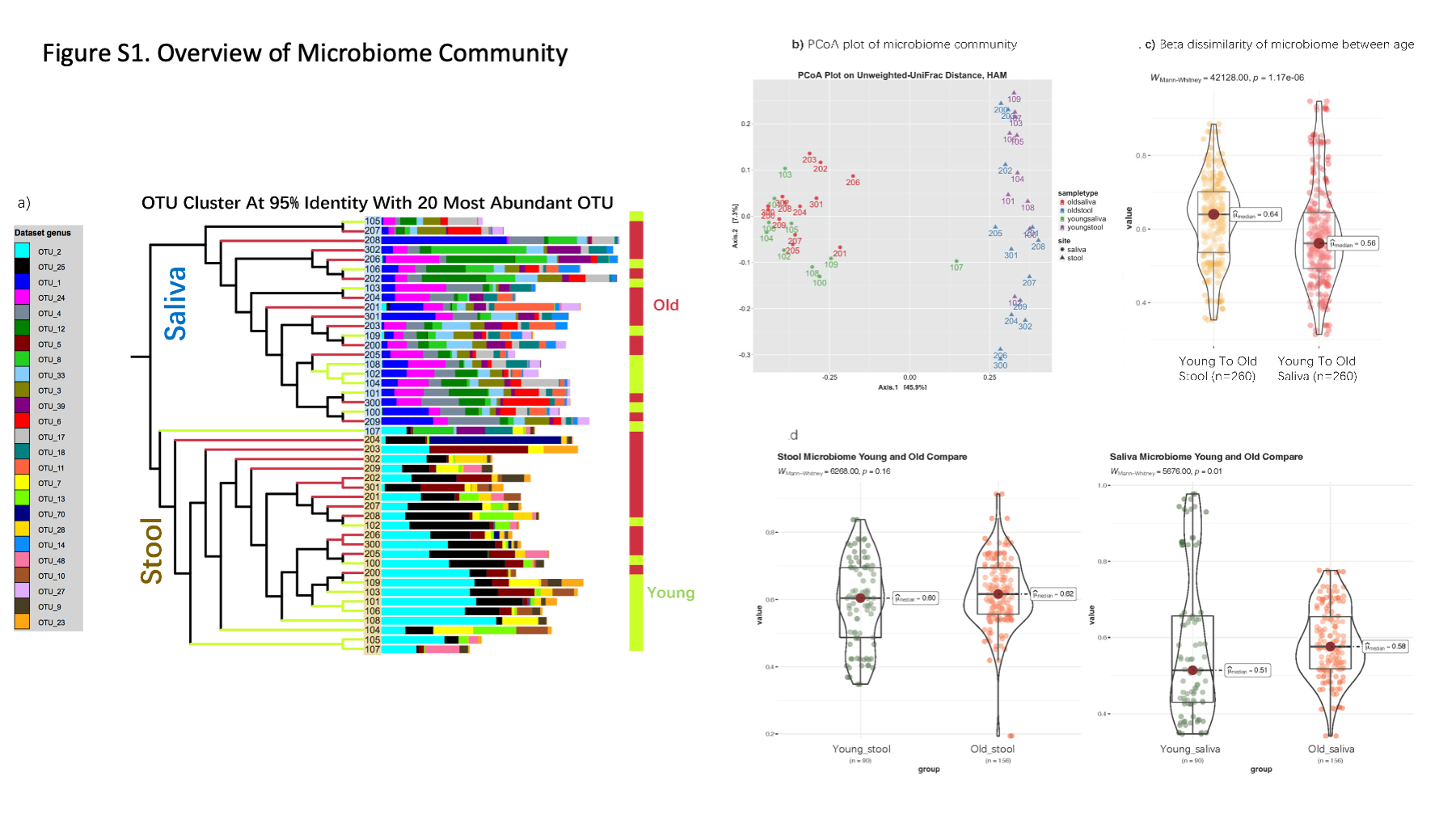

Supplement: Supplementary file 2 [file Image1.tiff]

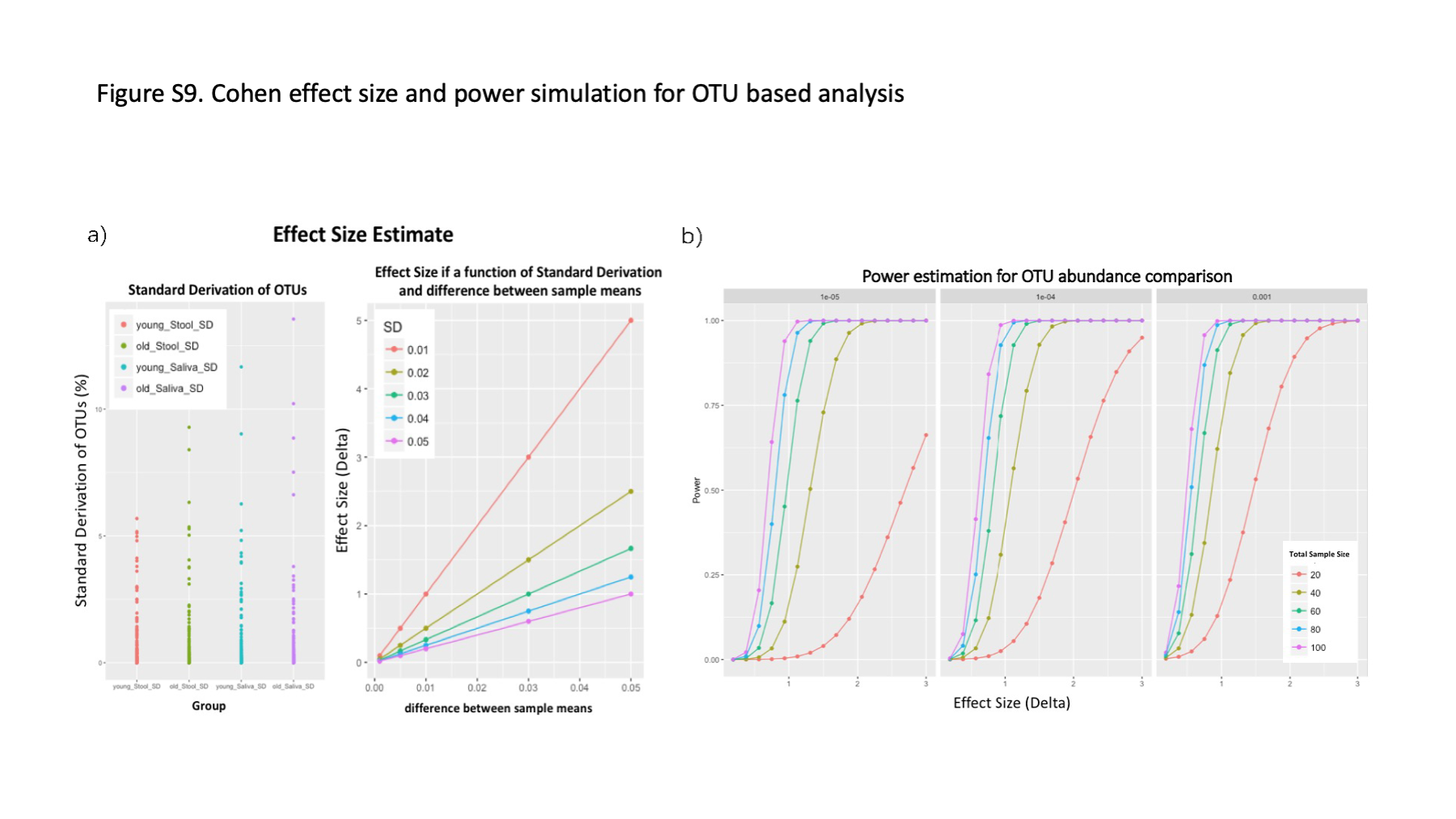

Supplement: Supplementary file 3 [file Image9.tiff]

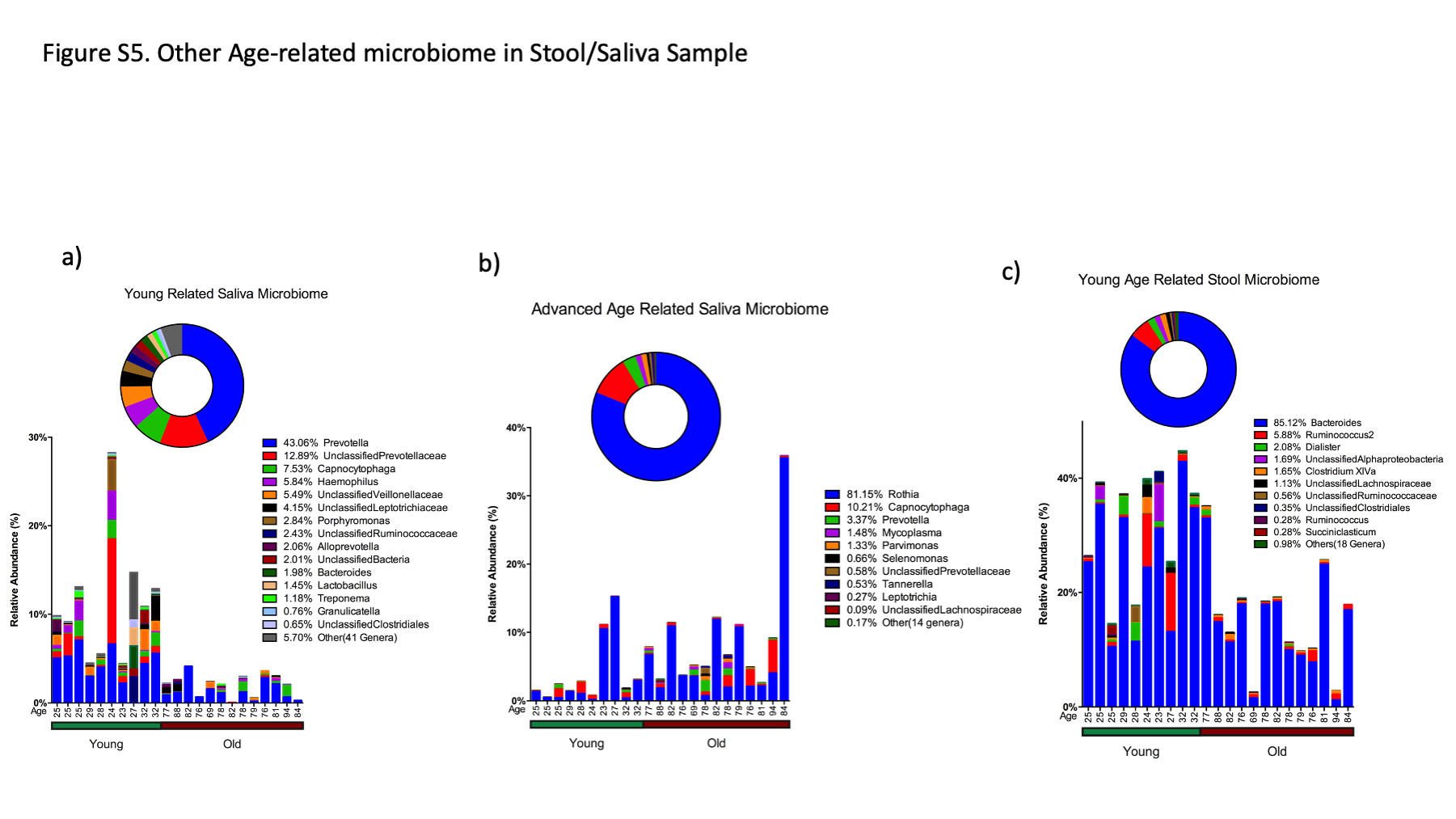

Supplement: Supplementary file 4 [file Image5.tiff]

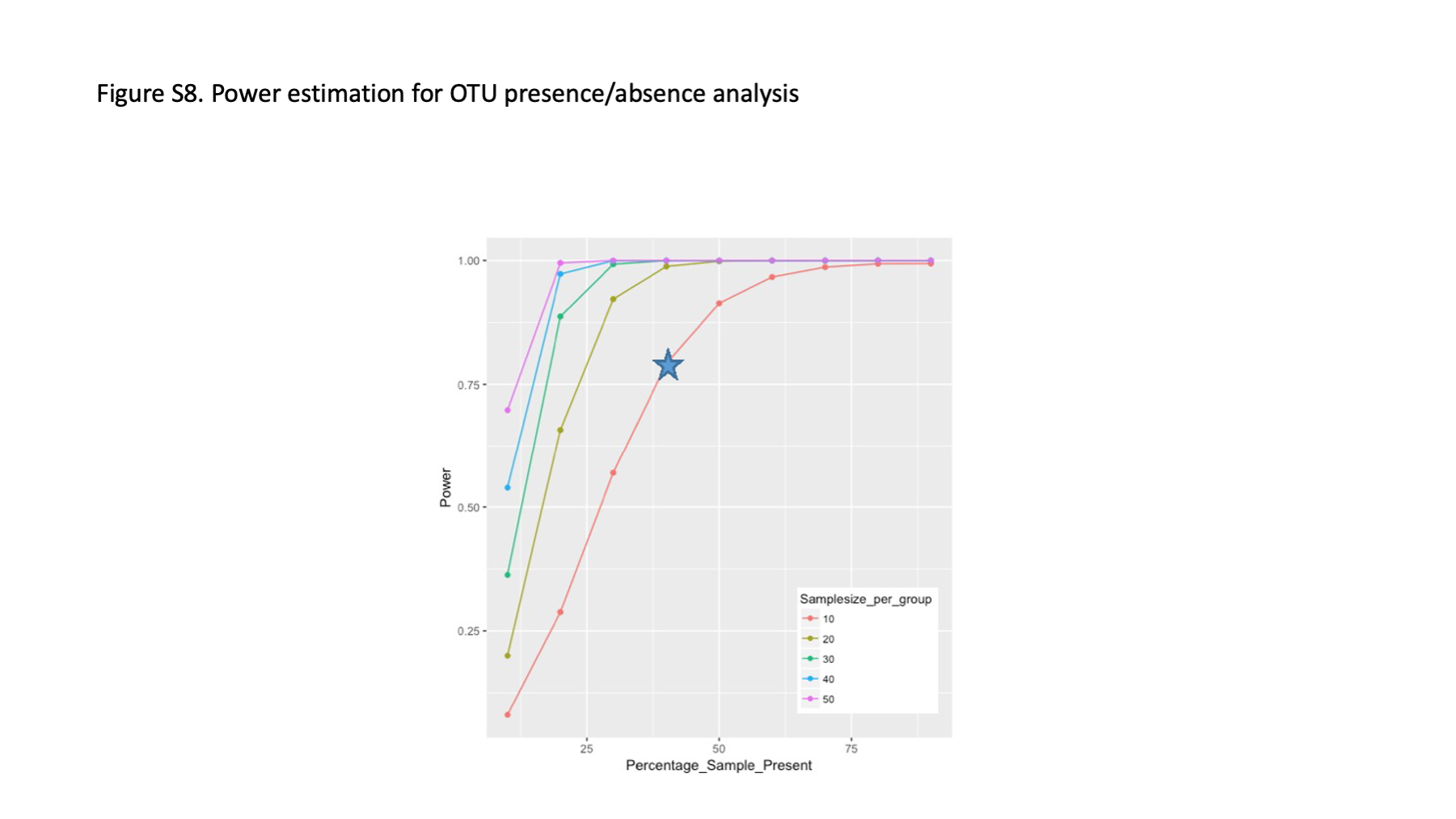

Supplement: Supplementary file 5 [file Image8.tiff]

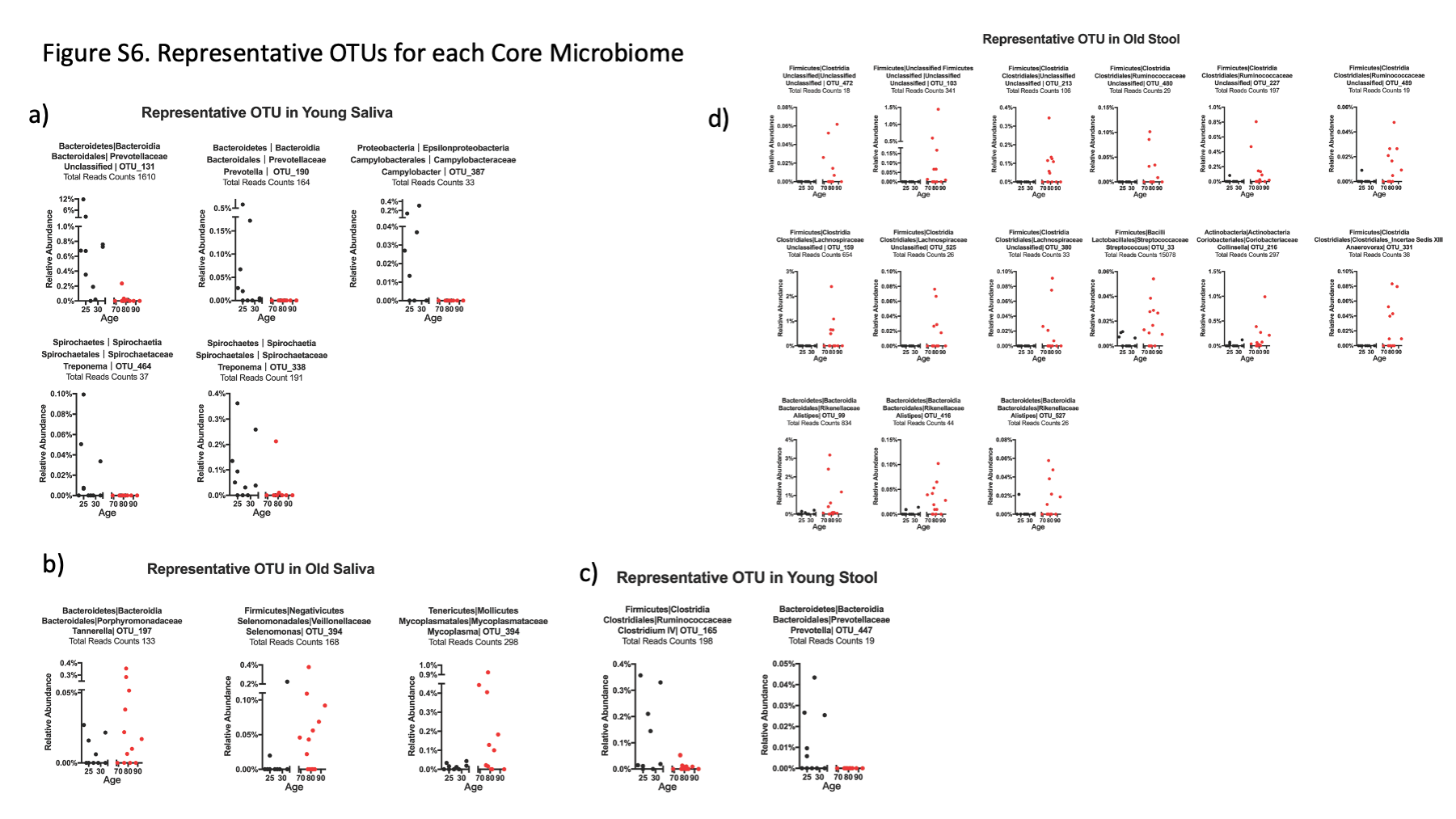

Supplement: Supplementary file 6 [file Image6.tiff]

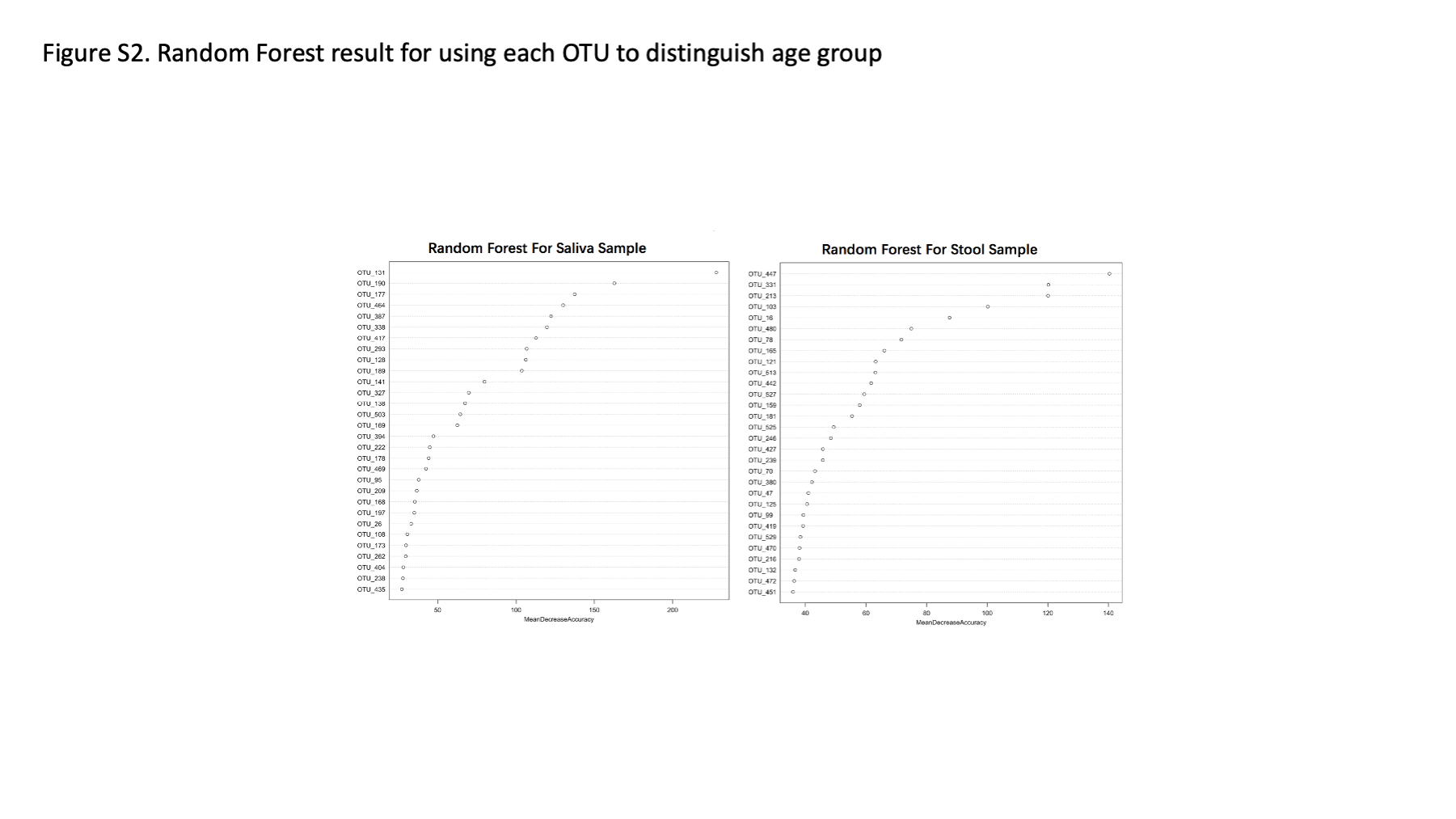

Supplement: Supplementary file 7 [file Image2.tiff]

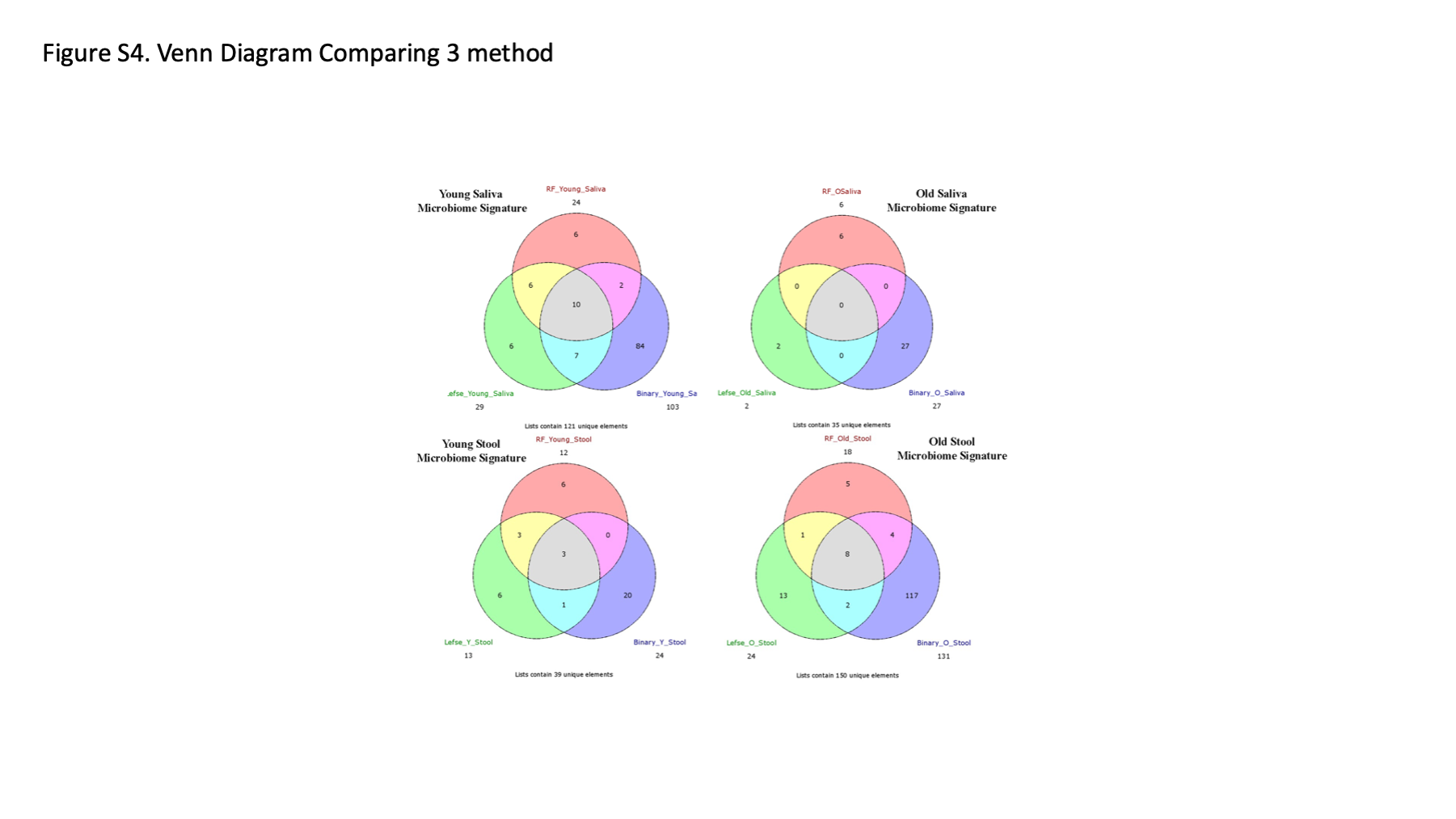

Supplement: Supplementary file 8 [file Image4.tiff]

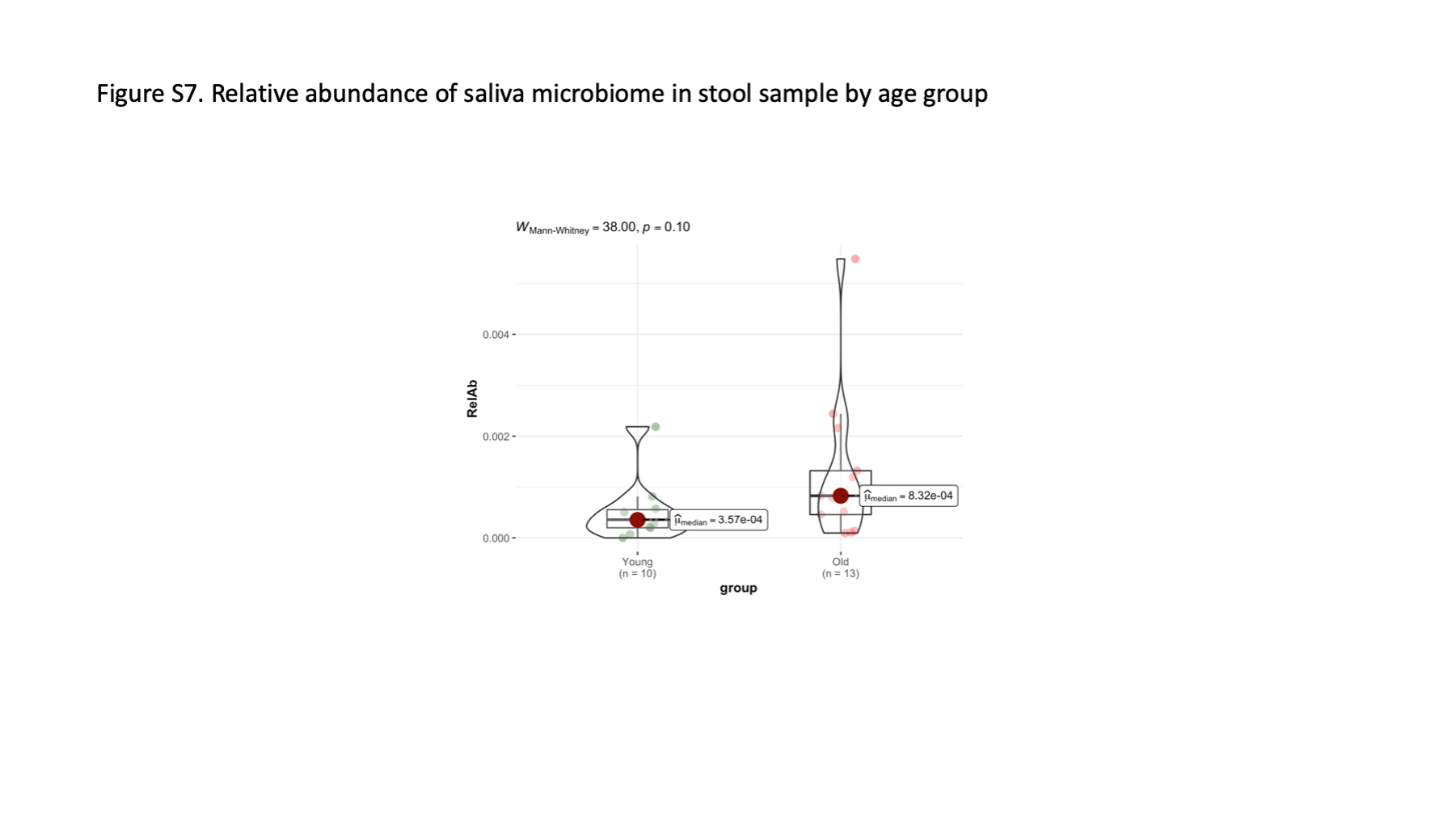

Supplement: Supplementary file 9 [file Image7.tiff]
